# A Comprehensive Review of Bioelectrical Impedance Analysis and Other Methods in the Assessment of Nutritional Status in Patients with Liver Cirrhosis

**DOI:** 10.1155/2017/6765856

**Published:** 2017-08-13

**Authors:** Halina Cichoż-Lach, Agata Michalak

**Affiliations:** Department of Gastroenterology, Medical University of Lublin, 20-094 Lublin, Poland

## Abstract

It is assumed that approximately 24–66% of patients with liver cirrhosis develop malnutrition. Numerous pathological processes lead to serious disorders of nutritional status in this group of patients. Malnutrition in the course of liver cirrhosis is associated with increased morbidity, complications, and low quality of life. Under these conditions, detection of malnutrition is of crucial importance. This review explores the complex mechanisms that lead to malnutrition in the course of liver cirrhosis and focuses on methods used in the assessment of nutritional status in cirrhotic patients. Among others, the role of bioelectrical impedance is highlighted. This noninvasive tool is promising and quite an accurate method of estimating body composition.

## 1. Malnutrition in the Course of Liver Cirrhosis and Methods of Nutritional Status Assessment—General Data

Nutritional assessment in patients with cirrhosis constitutes an extremely important issue in hepatology and has an undeniable impact on cirrhotic patients' outcome. Protein caloric malnutrition is described as the gradual loss of both lean body mass (protein) and adipose tissue (calorie), coexisting with the expansion of extracellular mass [1, 2]. Several pathomechanisms participate in the development of malnutrition in cirrhotic patients, for example, anorexia followed by inadequate oral ingestion, nausea, vomiting, early satiety, and side effects of administered drugs. Malnutrition is a commonly precipitating state among cirrhotic patients, and its prevalence might be even 80% [3]. Moreover, poor absorption (especially in patients with coexisting bile duct diseases) and increased gastrointestinal permeability are also involved [4]. In consequence, because of the deterioration in the storage of nutrients and altered hepatic synthesis, hypoalbuminemia occurs. It is further accompanied by hydroelectrolytic imbalance caused by renal insufficiency. Those pathological processes finally lead to the loss of organism homeostasis and various systemic disturbances [5, 6]. Malnutrition in cirrhotic patients is inseparably connected with poor quality of life, increased risk of infections and complications, presence of refractory ascites, hepatic encephalopathy, and even the first episode of bleeding from the gastrointestinal tract (GIT) [7–11]. Therefore, malnutrition appears to be a relevant factor when determining the progress of hepatic disease and it is the reason for its great importance. Researchers proved a correlation between malnutrition and mortality in a compensated group of patients, but these data deserve particular attention and further research [12]. Nevertheless, the identification of the best nutritional assessment method still remains a controversial topic, and the gold standard in this area has not been established yet [13, 14]. Several methods are performed to assess nutritional status of cirrhotic patients. The subjective global assessment (SGA) analyzes basic information obtained from a patient and physical examination. By means of dynamometry handgrip, strength is measured. Moreover, bioelectrical impedance is also used. The objective utilization of bioelectrical impedance analysis (BIA) and its accuracy among cirrhotic patients are still discussed. A lot of scientists suggest creating specific equations devoted to this group of patients because of their corporal alterations (e.g., ascites). This procedure should increase the effectiveness of BIA assessment. It has also been proven that phase angle (PA) can serve as a powerful indicator of survival and even correlate with the Child-Pugh score [15]. The accurate assessment of nutritional status among cirrhotic patients might lead to the improvement in their management and appropriate treatment [16, 17].

## 2. Metabolic Disorders in Cirrhotic Patients

### 2.1. Pathological Appearance of Hepatic Encephalopathy

Liver insufficiency leads to changes in body composition through the deterioration of the urea cycle followed by the accumulation of ammonia. Skeletal muscles become an important organ participating in ammonia metabolism under those pathological conditions. Due to this fact, there is growing evidence that the assessment of both muscle mass and muscle function is of crucial importance in cirrhotic patients. Additionally, one of the most severe complications of liver disorders—hepatic encephalopathy (HE)—might be exacerbated by nutritional status and ameliorated by nutritional treatment [18–20]. On the other hand, neurocognitive disturbances in patients with HE not rarely make it impossible to accomplish reliable results of performed nutritional status tests. Therefore, trustworthy strategies are required to evaluate the body composition of patients with hepatic encephalopathy [21]. The role of nutrition in the development of HE is well known, and nutritional treatment has an impact on the quality of life together with the survival of cirrhotic patients. The most appropriate management in those patients should embrace practical nutritional strategies together with understanding all the pathological processes involved in HE [22–24].

### 2.2. Role of Amino Acids

Subsequently, the beneficial role of branched-chain amino acids (BCAAs) in the management of HE is currently highly emphasized. A lower concentration of the key BCAAs (valine, leucine, and isoleucine) has been diagnosed in cirrhotic patients. Nutritional management based on BCAA supplementation constitutes an element of treatment for cirrhosis and HE. Pathogenesis of BCAA decrease is explained by the false transmitter hypothesis. A pivotal issue in this theory is imbalance in the blood amino acids, resulting in increased efflux of cerebral aromatic amino acids. The final stage of this cascade is the release of false neurotransmitters. Furthermore, the level of BCAAs is decreased due to the activated glutamine synthesis in the muscle [25–27]. Simultaneously, the muscular uptake of BCAAs increases [28]. Another pathological aspect of BCAAs is their influence on muscle metabolism, which is reflected by glutamine synthesis from ammonia fixation together with inhibition of proteolysis. According to the newest findings, BCAA supplementation improves the nourishment of cirrhotic patients. It might also lead to an increase in ammonia, although coexisting exacerbation of HE was not observed. Other studies indicate a beneficial role of BCAAs in ameliorating HE manifestations [29–33].

### 2.3. Ammonia Toxicity

Ammonia is still widely described as a crucial toxin responsible for neurological impairment in cirrhotic patients. Nevertheless, blood ammonia concentration does not strictly correlate with the severity of HE in all cases and should always be assessed with reference to the clinical state. We can distinguish three main pathways of ammonia metabolism: through the intestines, kidneys, and muscles. The liver is a major organ responsible for ammonia clearance in normal circumstances, and the skeletal muscles play a secondary role in this process. The intestinal clearance of ammonia remains a key issue, as the colon constitutes one of the most important treatment targets in the course of HE [34, 35]. Lactulose and lactitol belong to the best-known nonabsorbable disaccharides. Apart from the fact that lactulose is a laxative, conducted studies proved its role in the conversion of ammonia to ammonium by the formation of acetic acid through lactic acid. What is interesting, colon acidification promotes the development of nonammoniagenic bacteria. Because of this fact, the prebiotic activity of disaccharides could be enhanced by nutritional treatment [36]. Since hemodynamic disturbances can occur in cirrhotic patients, kidney production of ammonia is elevated. Subsequently, because of liver failure and a disrupted urea cycle, ammonia is accumulated in the brain tissue and skeletal muscles, where glutamine synthetase can be found, an enzyme that transforms ammonia and glutamate into glutamine. Glutamine is an example of osmocyte and causes the movement of water into the cells. This phenomenon is especially harmful among astrocytes leading to intracranial hypertension together with cerebral edema. Therefore, the role of muscle mass in the detoxification of ammonia becomes an incredibly important issue in cirrhosis [25, 37].

### 2.4. Muscles in the Course of Liver Cirrhosis

Nearly half of the total ammonia might be metabolized in the muscles by glutamine synthesis. According to research results, this process is not so efficient in the rest state; however, it could be more important in cirrhotic patients. Thus, a lot of studies postulated that physical activity (e.g., 5000 steps daily) could be a good solution for cirrhotic patients to improve ammonia metabolism. Muscle wasting might concern even 45% of cirrhotic patients [38, 39]. Interestingly, scientists found a correlation between fat-free mass (FFM) and albumin level in the course of active cirrhosis. This result suggests that muscle volume could be the crucial element in maintaining the serum albumin level in cirrhotic patients [40, 41]. Liver cirrhosis belongs to catabolic diseases, and final stage cirrhotic patients are usually affected by muscular wasting, which attenuates the conversion of ammonia to glutamine. The abovementioned mechanism is mainly caused by deteriorated glucose metabolism that leads to the use of muscular proteins as an energy source in a fasting state. What is more, cirrhotic patients tend to develop BCAAs. Because of the enumerated mechanisms, a proper assessment of nutritional status and muscle mass is crucial in each stage of the disease in cirrhotic patients in order to introduce an appropriate dietary treatment and reduce the process of muscle wasting [42–44].

### 2.5. Alterations in the Gastrointestinal Tract

An inflammatory process is also involved in the pathological appearance of cirrhosis. Proinflammatory cytokines cross the blood-brain barrier and facilitate the ammonia diffusion into astrocytes. Conducted studies revealed elevation in fecal calprotectin in cirrhotic patients suggesting that GIT is the main source of inflammation in this group of patients. Slow gastrointestinal transit and mucosal edema in the course of cirrhosis have been reported to allow bacterial products to force the epithelial barrier and finally reach the portal vein. At the end of this cascade, the release of tumor necrosis factor occurs, causing further damage to the organism. In addition, small bacteria overgrowth is observed in GIT, leading to disturbances in the microbiota profile. Performed surveys proved that nutritional treatment based on probiotics, prebiotics, symbiotics, and fermentable fiber may limit this process and improve GIT transit and the composition of bacterial flora [45]. Electrolyte imbalance is another significant factor participating in liver cirrhosis. Hyponatremia is widely agreed to be the third hit in the course of HE because of elevating the blood-brain barrier permeability and exacerbating of HE. Moreover, not rarely, coexisting hypokalemia might increase ammonia production and excretion by the kidneys. High serum manganese concentration resulting from impaired liver function, altered bile flow, and the presence of portosystemic shunts are also observed in cirrhotic patients. According to conducted investigations, manganese tends to accumulate in the brain tissue; it was detected in the caudate nucleus and in the globus pallidus of patients with severe HE. This detection might explain the presence of selected Parkinson's symptoms (such as tremors) observed in certain patients with HE. Moreover, manganese is the first metal reported to induce apoptosis in the central nervous system [46]. Dietary proteins are a substantial source of zinc. Depletion in this element in cirrhotic patients is reflected by diarrhea, muscle cramps, skin lesions, and even HE. Low levels of trace elements, selenium, copper, calcium, iron, and magnesium, are also a concern. Nevertheless, according to the newest data, it is impossible to correlate the degree of their deficiency with the progress of cirrhosis [47]. Small intestinal bacterial overgrowth (SIBO) is another phenomenon participating in the pathological appearance of liver diseases, especially alcoholic cirrhosis. Its prevalence was proved to correlate with the degree of malnutrition according to SGA. There are several reasons responsible for this state. First of all, SIBO constitutes a factor leading to the inflammation of the intestinal mucosal membrane. Due to this fact, bacteria are able to invade the bowel and interrupt metabolic processes. As a result, the consumption of carbohydrates, fat-soluble vitamins, and proteins increases. Additionally, bacterial deconjugation of bile salts occurs. All the enumerated factors finally lead to malabsorption. SIBO is also inseparably connected with pathological symptoms from GIT, for example, abdominal pain, diarrhea, and increased flatus [48]. Vitamin A deficiency, a common disorder observed among cirrhotic patients, is also indirectly connected with SIBO [49]. What is more, dysbacteriosis and hyperammonemia are inseparable phenomena, responsible for the development of neuroinflammation in cirrhotic patients. Therefore, SIBO can become another target for the treatment of cirrhosis [50, 51]. Taking into consideration all the above-discussed mechanisms participating in malnutrition of cirrhotic patients, it is obvious that the assessment of nutritional status is of crucial importance in each stage of the disease. There is also a great demand for reliable and comprehensive tools to evaluate nutritional status. According to the newest data, it has been established that 75% of cirrhotic patients with encephalopathy will develop moderate to severe malnutrition. Because of its muscular role in ammonia detoxification, malnutrition is inseparably related to a higher risk of HE. Malnutrition in cirrhotic patients is protein caloric, leading to both lean and fat tissue depletions. Simultaneous reduction in those tissues is commonly known as cachexia. Nevertheless, it is sarcopenia, which is manifested by the first result of nutritional deficiency in the course of cirrhosis [52–54]. On the other hand, being overweight has been described as another important phenomenon among cirrhotic patients and called as sarcopenic obesity, reflected by excess adipose tissue and muscle wasting. Interestingly, some data imply the obesity paradox in the course of cirrhosis, suggesting that lower mortality in this group of patients is inseparably connected with a higher nutritional reserve [55]. Nutritional assessment of cirrhotic patients must involve several areas: well-taken dietary history, body composition, and laboratory tests [56]. Advantages and disadvantages of commonly used methods in the assessment of nutritional status among cirrhotic patients are shown in Table 1.

## 3. Nutritional Assessment of Cirrhotic Patients

### 3.1. Subjective Global Assessment

SGA is one of the most common scales used to evaluate nutritional status in patients during their hospital stay. It includes data concerning food intake, weight changes, symptoms from GIT, and physical examination to evaluate subcutaneous fat, muscular atrophy, ascites, and edema. All the results are grouped into three categories from A to C, where C means severe malnourishment [57]. It is a useful screening method performed at the admission to a hospital; however, in cirrhotic patients, its usefulness is limited by several issues. Firstly, SGA depends on personal information that can be impossible to obtain from a patient with neurocognitive disturbances. Furthermore, the only anthropometric measure used in the scale is weight which might be affected by ascites and edema. SGA is not recommended in cirrhotic patients because it may underestimate malnutrition and does not predict outcome accurately [58]. Nevertheless, performed research revealed the correlation between malnutrition assessed by SGA and mortality in the course of cirrhosis [59].

### 3.2. Anthropometric Measurements

Anthropometric measurements are objective, noninvasive, rapid, and low-cost tools evaluating nutritional status, dedicated especially to the assessment of somatometric characteristics. Anthropometry is said to be the most useful method in evaluating malnutrition in cirrhotic patients. However, it is influenced by ascites and edema and the final measurement of body weight and BMI might be underestimated. There is a special BMI classification devoted to cirrhotic patients, but it is not commonly used in clinical trials and does not have a clear single cutoff value to diagnose malnutrition. A predominant number of surveys still use the dry weight, which is measured by subtracting the weight of edema and ascites according to the information gathered during clinical assessment. It is worth highlighting the use of triceps skinfold (TSF) and midarm muscle circumference (MAMC). MAMC is calculated from TSF and midarm circumference (AC). It is considered to be the most sensitive marker of BCM [60, 61]. Furthermore, a decrease in both TSF and MAMC was discovered to correlate with the severity of cirrhosis according to the Child-Pugh score [62]. Other authors also suggest the use of corrected arm muscle area (CAMA), which is calculated from AC and TSF values. Several investigations proved their accuracy in combination with SGA. What is worthy of note, another group of scientists found a more significant affection of MAMC values in women and higher loss of deposits based on TSF in men, which may lead to discrepancies between genders. In general, muscle mass is physiologically higher in men and the proportion of muscle wasting is higher, too [63]. Adductor pollicis muscle thickness (APMT) is a following parameter helpful in the diagnosis of muscle wasting in cirrhotic patients. The aforementioned parameters belong to relatively powerful and reliable tools because their measurement is not influenced by ascites and edema. Nonetheless, even these parameters do not have established cutoff values in the population of cirrhotic patients. Therefore, anthropometric results should be confirmed by other methods to achieve the highest exactness in nutritional status assessment [64].

### 3.3. Bioelectrical Impedance Analysis

Bioelectrical impedance analysis (BIA) measures belong to safe, rapid, easy-to-perform, and quite accurate methods of estimating fat mass and fat-free mass. According to their basic assumption, the human organism behaves like a constant cylinder with unchanging conductivity. Thanks to this rule, empirical estimation of body water and body composition might be obtained. The water component of the body is estimated with regard to the capacity of the body to conduct an electrical current. Because of this fact, the main assumption of BIA is that electrical conduction has a greater speed through water and electrolyte-containing parts than through fat tissue and bone mass. This happens due to the resistance exerted by fat deposits together with bones. A fixed, low-voltage, high-frequency alternating current is introduced into the human body to perform BIA and assess body electrical conductivity together with resistance (impedance). Subsequently, capacitance is the parameter that makes the current lag behind the voltage, which results in a phase shift. This shift is measured geometrically as the angular transformation of the ratio of capacitance to resistance, or the phase angle (PA) [65–68]. PA is the determinant of the relative contribution of fluid (resistance) and cellular membranes (capacitance) of the human body. It is positively correlated with capacitance and negatively correlated with resistance [69, 70]. PA also reflects water distribution between extra- and intracellular spaces, and the altered distribution is a well-known indicator of malnutrition. This supports the idea that PA is an indicator of cell mass and nutritional risk. High phase angle score reflects a proper cellular membrane function, while lowered phase angle is characteristic of both decreased cellular matrix component and apoptosis of cell [71, 72]. Inappropriate values of PA characterize pathological states where the cell membrane integrity and fluid balance are impaired. There are several parameters that can be measured by means of BIA: body cell mass (BCM), total body water (TBW), extracellular water (ECW), extracellular mass (ECM), and body fat (BF) [73, 74]. Interestingly, several performed investigations proved a correlation between the decrease in PA and the development of hepatic encephalopathy in the course of liver cirrhosis [21]. Recently, the role of multifrequency BIA (MF-BIA) analysis has been emphasized because it is less influenced by overhydration. It measures the abovementioned parameters by passing a series of different electrical currents and electrical frequencies through the body. Assessments are performed in the supine position, with the use of an eight hand and feet tactile electrode system [75]. Segmental BIA is also used to overcome fluid retention in patients. As opposed to MF-BIA, single-frequency BIA (SI-BIA) might cause the underestimation of body composition [76]. It is usually performed with four electrodes. PA values are reported to be decreased in the course of advanced cirrhosis. What is more, scientists discovered a correlation between muscle strength and muscle mass in cirrhotic patients. Interestingly, a PA result less or equal than 5.4 degrees was a predictor of reduced survival in one of the conducted surveys [77, 78]. The data obtained from one of the latest investigations indicated PA cutoff less than or equal to 4.9 as a predictor of disease progression and mortality in cirrhotic patients [79]. BCM stands for a lean tissue compartment and is significantly decreased in protein-calorie malnutrition, such as in cirrhosis [80]. It makes it possible to assess protein turnover and energy expenditure. Several analyses demonstrated a correlation between hypermetabolism and coexisting low BCM proportion (below 35% of body weight) and short survival. Such a relationship was even proved in cirrhotic patients after transplantation [81]. Additionally, another survey revealed a decrease and a negative correlation between BCM and the model for end-stage liver disease (MELD) score in cirrhotic patients [82]. BIA analysis remains a relatively controversial tool in assessing nutritional status because of its several limitations, for example, fluid retention, diuretic use, liquid and food intake before the test, physical activity, and BMI value. All those numerous issues might underestimate the final result of this method. Unfortunately, they are common in the majority of cirrhotic patients. First of all, fluid overload might be the source of imprecision in BIA results. The increased ECW to TBW ratio is one of the most characteristic features in decompensated patients with ascites. What is more, PA results might be influenced by abnormal physical structure. A pacemaker or implanted cardiac defibrillator and amputation in medical history also belong to exclusion criteria before performing BIA [77]. Body positioning during the procedure, skin temperatures, abstinence from alcohol and caffeine, ambient air, and voiding prior to the measurement can also affect BIA results [83]. Several studies revealed no correlation between BIA results compared to SGA results or dual-energy-X-ray absorptiometry (DEXA). Nevertheless, the association between CAMA, MAMC, handgrip strength, and BCM was established. According to the other data, chest and abdomen together represent approximately 11% of the body resistance, indicating that BCM obtained from BIA was associated with the same measure gained from total body potassium count, independently of the presence of ascites. It is worth noticing that about 72% of participants in the research used diuretics. Another relevant fact is that ascites in cirrhosis constitutes only an element of diffuse water retention. The infusion of peritoneal cavity with 2-3 liters of fluid has a marginal effect on total body resistivity, fat mass, and fat-free mass, as it was observed in patients submitted to peritoneal dialysis. Furthermore, subsequent study demonstrated that removal of ascites affects BCM values only to a small extent [73]. Nevertheless, there is still a great demand for verifying the precision of BMI measurements in the course of liver cirrhosis and for correlating them with, for example, the Child-Pugh score, because data obtained in this are still obscure [60]. Additionally, among BIA parameters, PA was observed to be less affected by overhydration while being a reliable indicator of clinical outcome. A survey conducted on critically ill patients, gastroenterological patients, among others, demonstrated a statistically significant association between the PA value and the albumin level and a negative correlation between ECW/TBW and albumin. The same study has shown that lower PA together with higher ECW/TBW are characteristics of nonsurvivors [81]. Because of its simplicity, BIA could be a common meaningful tool not only in the evaluation of malnutrition but also in a follow-up in a great number of patients [77]. Some data have suggested that PA can be a potential predictor of prognosis in chronic obstructive pulmonary disease, hemodialysis, human immunodeficiency virus infection, sepsis, liver cirrhosis, and lung, colorectal, and pancreatic cancer [60, 66, 84–88]. Although BIA is the source of prognostic information about cirrhotic patients, it still requires further investigation to assess its precision. BIA might serve an important role in palliative care by predicting the outcome of terminal stage patients and avoiding unnecessary treatment [71]. Various investigations revealed the correlation between phase angle and survival in the course of terminal gastrointestinal system cancers (stomach, colon, and liver cancer). According to existing data, PA score below 5 degrees reflects a poor outcome and due to this fact has a prognostic value in assessing survival in terminal cancer patients. It could be also helpful in choosing the most appropriate therapy in the course of advanced cancer. Nevertheless, a certain PA cutoff value has not been established yet. Ischemia and reperfusion (I/R) injury in the liver constitutes another field of BIA possible usefulness. Several investigations demonstrated the role of BIA assessment during liver surgery. Despite liver tolerance to ischemia, hepatocytes after reperfusion might become damaged. With the use of conventional methods, it is usually impossible to monitor liver parameters during surgery. BIA is the tool which might be used in a real-time manner in the course of liver surgery. What is more, the results may be compared with BIA parameters assessed before and after the surgery to determine liver function status. One of performed surveys conducted on rats revealed a statistically significant increase in BIA values at low-frequency electric current during ischemia. Furthermore, according to results of the study, reperfusion made BIA parameters return nearly to the baseline level, which has been observed to a greater extent in rats with shorter periods of ischemia [89]. Liver transplantation is still the matter of crucial importance because of its influence on nutritional status. BIA is also helpful in this area to determine body composition and predict protein-energy malnutrition after liver transplantation [90, 91]. Some data have suggested the potential role of BIA in predicting posttransplant outcomes; however, this issue should be clarified in further investigations. Not only BIA measurements but also aforementioned SGA TSF and the degree of sarcopenia might serve an important role in the assessment of nutritional status and health-related quality status after liver transplantation. Because of the crucial role of the liver in the human organism and its implications on numerous processes, this field should be undoubtedly explored [92–94].

### 3.4. Muscles Assessment

Muscles' activity assessment appears to be a matter of great importance because of their role in ammonia metabolism in cirrhotic patients. Tests requiring extensive collaboration cannot be applied in population suffering from HE. Thus, handgrip strength (HS) might be a solution for patients with low-grade HE to evaluate nutritional status. During this procedure, the strength exerted by a nondominant hand is measured with a dynamometer [15]. It has been reported as a simple and valuable tool by several guidelines in accomplishing cirrhosis decompensation. However, a significant limitation of this method was revealed—it depends on sex. Researchers found that the level of muscle loss in the course of liver cirrhosis is different in each gender and skeletal muscle function is correlated with muscle mass only in men [95].

Dual-energy-X-ray absorptiometry (DEXA) examination is based on the passage of photons through certain elements of the body (bone, fat, lean, and bone-free lean mass). Radiation exposure is minimal with this technique. DEXA estimate allows to accomplish lean mass and fat-free mass which technically differ from each other. Fat-free mass includes nonfat constituents of the body together with a small amount of adipose tissue. Lean mass consists of nonadipose elements, which contain the lipids gathered in nervous tissue and in cell membranes. DEXA is commonly used to validate the results of body composition performed by other procedures [96]. It is worth mentioning that numerous authors postulated its high reproducibility and a relatively good correlation between total body fat estimated from anthropometry and DEXA even in patients with ascites. Nonetheless, there are also discrepancies between men and women in DEXA, resulting from different characters of soft tissue loss. In cirrhotic women, more reduction in fat stores is observed with the maintenance of lean tissue, as in early starvation. In men, the loss of lean tissue is the most featured phenomenon that resembles the state under the stress. A described pattern is reflected by weak association between muscle strength and muscle mass in cirrhotic women, which was mentioned above. It is also possible to appraise the fat-free mass index (FFMI) with the use of DEXA, which means the ratio of the lean mass to the square of the height [97]. In the survey of cirrhotic patients enrolled for liver transplantation, this parameter demonstrated a correlation between muscle wasting and the development of HE. There is also growing evidence that DEXA might be used to evaluate the degree of sarcopenia, reflected by the appendicular muscle mass index (AMMI), calculated by dividing the sum of appendicular muscle mass index by the square of the height. This approach is confirmed by several studies dedicated to cirrhosis which proved that AMMI results in cirrhotic patients are not affected by fluid retention or overweight.

The role of the computed tomography (CT) scan in assessing cirrhosis-related lean mass waste is also emphasized in literature nowadays. What is interesting, this procedure enables to an appraisal of abdominal muscles which are usually inaccessible. Furthermore, thanks to a CT scan, the measurement of transversal muscle thickness is possible. Several scientists have suggested performing images targeted on the third lumbar vertebra because this procedure makes it possible to assess whole body fat and fat-free mass. The psoas muscle index has also been proved to correlate with clinical parameters and the degree of skeletal muscle mass loss in cirrhotic patients. Nevertheless, the skeletal muscle index at the third lumbar vertebra is described as the most reliable single imaging which correlates to the whole body muscle mass [15, 42, 98–102].

## Figures and Tables

**Table 1:** Methods to assess nutritional status in cirrhotic patients with their advantages and disadvantages.

*SGA*	*Anthropometry*
(i) Quick application and low cost (ii) Can be obtained in hospital rooms (iii) Identifies patients under risk of malnutrition upon hospital arrival (iv) Requires patient comprehension and collaboration (v) Subjective method (vi) Weight is the only objective measurement (vii) Might underestimate malnutrition	(i) Low cost (ii) Can be obtained in hospital rooms (iii) Requires little collaboration (iv) CAMA, MAMC, APMT less influenced by water retention/overweight (v) MAMC was investigated to correlate with Child-Pugh score (vi) Some indices (body weight, body mass index, AC, TSF) might be influenced by water retention/overweight

*Handgrip strength*	*BIA*
(i) Quick application and low cost (ii) Can be obtained in hospital rooms (iii) Indicates impaired muscle function (iv) Is not influenced by water retention/overweight (v) Cannot identify muscle wasting anatomically (vi) Is suitable only in men, because skeletal muscle function correlates with muscle mass only in men (vii) Requires patient comprehension and collaboration	(i) Quick application (ii) Can be obtained in hospital rooms if portable (iii) PA and BCM correlate with outcomes in cirrhotic patients (iv) Controversial application in patients with fluid retention (v) Can underestimate malnutrition

*DEXA*	*CT scan*
(i) Indicates muscle depletion accurately with high reproducibility (ii) Detailed assessment of body composition (segmental results) (iii) AMMI reflects sarcopenia and is not influenced by fluid retention/overweight (iv) High cost (v) Exposure to ionizing radiation (vi) Requires patient removal to the equipment room	(i) Indicates muscle depletion accurately with high reproducibility (ii) Skeletal muscle index at the third lumbar vertebra correlates with the whole body muscle mass and might be used to diagnose sarcopenia (iii) High cost (iv) Exposure to ionizing radiation (v) Requires patient removal to the equipment room
